# Accuracy of a rapid glial fibrillary acidic protein/ubiquitin carboxyl‐terminal hydrolase L1 test for the prediction of intracranial injuries on head computed tomography after mild traumatic brain injury

**DOI:** 10.1111/acem.14366

**Published:** 2021-09-07

**Authors:** Jeffrey J. Bazarian, Robert D. Welch, Krista Caudle, Craig A. Jeffrey, James Y. Chen, Raj Chandran, Tamara McCaw, Saul A. Datwyler, Hongwei Zhang, Beth McQuiston

**Affiliations:** ^1^ Departments of Emergency Medicine and Neurology University of Rochester Rochester New York USA; ^2^ Department of Emergency Wayne State University School of Medicine Detroit Michigan USA; ^3^ Biostatistics and Epidemiology Research Design Core Wayne State University Detroit Michigan USA; ^4^ Warfighter Brain Health Project Management Office US Army Medical Materiel Development Activity, US Army Medical Research and Development Command Fort Detrick Maryland USA; ^5^ Abbott Point of Care Ottawa Ontario Canada; ^6^ Department of Radiology San Diego Veterans Administration Medical Center La Jolla California USA; ^7^ Department of Radiology UC San Diego Health System La Jolla California USA; ^8^ Abbott Diagnostics Abbott Park Illinois USA

## Abstract

**Objective:**

The objective was to determine the accuracy of a new, rapid blood test combining measurements of both glial fibrillary acidic protein (GFAP) and ubiquitin carboxyl‐terminal hydrolase L1 (UCH‐L1) for predicting acute traumatic intracranial injury (TII) on head CT scan after mild traumatic brain injury (mTBI).

**Methods:**

Analysis of banked venous plasma samples from subjects completing the Prospective Clinical Evaluation of Biomarkers of Traumatic Brain Injury (ALERT‐TBI) trial, enrolled 2012–2014 at 22 investigational sites in the United States and Europe. All subjects were ≥18 years old, presented to an emergency department (ED) with a nonpenetrating head injury and Glasgow Coma Scale score (GCS) 9–15 (mild to moderate TBI), underwent head CT scanning as part of their clinical care, and had blood sampling within 12 h of injury. Plasma concentrations of GFAP and UCH‐L1 were measured using i‐STAT Alinity and TBI plasma cartridge and compared to acute TII on head CT scan.

**Results:**

Of the 2011 subjects enrolled in ALERT‐TBI, 1918 had valid CT scans and plasma specimens for testing and 1901 (99.1%) had GCS 13–15 (mTBI), for which the rapid test was intended. Among these subjects, the rapid test had a sensitivity of 0.958 (95% confidence interval [CI] = 0.906 to 0.982), specificity of 0.404 (95% CI = 0.382 to 0.427), negative predictive value of 0.993 (95% CI = 0.985 to 0.997), and positive predictive value of 0.098 (95% CI = 0.082 to 0.116) for acute TII.

**Conclusions:**

A rapid i‐STAT–based test had high sensitivity for prediction of acute TII, comparable to lab‐based platforms. The speed, portability, and high accuracy of this test may facilitate clinical adoption of brain biomarker testing as an aid to head CT decision making in EDs.

## INTRODUCTION

### Background

Head computed tomography (CT) remains the imaging modality of choice for the diagnosis of intracranial injury such as hemorrhage or edema for patients with mild traumatic brain injury (mTBI) treated in the emergency department (ED) during the acute postinjury period.^1^ CT scan along with patient symptoms and physical findings are the main factors used to direct care of such patients.^2^ It has long been suggested that CT head imaging for mTBI patients is overutilized in those patients at very low risk for acute intracranial hemorrhage.^3^ This concept, in conjunction with factors such as radiation exposure,^4^ excess ED resource usage, cost,^5^ and others, has prompted a search for tools that can effectively and safely identify patients at very low risk for intracranial abnormalities and, therefore, not needing CT imaging.

Clinical decision rules (CDR) that utilize easily obtained patient historic and physical findings have been developed in attempt to reduce CT use in low‐risk mTBI patients.^6^, ^7^ Despite considerable enthusiasm for CDRs, they have been difficult to widely implement^3^, ^8^, ^9^ and possibly are less sensitive than originally described.^10^

### Importance

The results of the ALERT‐TBI trial suggested that a test combining glial fibrillary acidic protein (GFAP) and ubiquitin carboxyl‐terminal hydrolase L1 (UCH‐L1; brain trauma indicator [BTI]) done within 12 h of head injury could reduce unnecessary head CT scanning by 30%,^11^ and data from this trial were used to support Food and Drug Administration (FDA) clearance for this test in February 2018. The BTI, when used in patients with mTBI in whom a head CT scan is felt to be clinically indicated, can, with a high degree of certainty, identify those at low risk for acute traumatic intracranial injury (TII). The 4 h required to perform the lab‐based BTI test, however, has precluded widespread clinical adoption for ED patients given the average wait time of over 3 h for a head CT scan in the ED.^12^, ^13^ The recent development of a rapid GFAP/UCH‐L1 test measured on the i‐STAT Alinity platform overcomes this hurdle.^14^, ^15^ The i‐STAT TBI test measures both GFAP and UCH‐L1 simultaneously and received FDA clearance on January 8, 2021.

### Goals of this report

Our study's main objective was to determine the predictive accuracy of a test combining both GFAP and UCH‐L1 measured using the i‐STAT Alinity and TBI plasma cartridge among those with mTBI by analyzing banked plasma samples from the prospective ALERT‐TBI trial and comparing results to the standard head CT scan. A secondary preplanned objective was to determine predictive accuracy among the following TBI subsets: those with a Glasgow Coma Scale (GCS) score of 14–15, a GCS of 15, and a GCS of 15 undergoing blood draw within 2 h of injury.

## METHODS

### Study design and setting

This is a secondary analysis of banked venous plasma samples from subjects successfully completing Prospective Clinical Evaluation of Biomarkers of Traumatic Brain Injury (ALERT‐TBI) trial.^11^ GFAP and UCH‐L1 were measured in available plasma samples using a rapid test, which were combined into a single test result that was compared to head CT results. The study design and reporting followed the STARD guidelines for diagnostic accuracy.^16^

### Selection of participants

Briefly, the ALERT‐TBI study prospectively enrolled 2011 acute TBI patients between 2012 and 2014 at 22 investigational sites globally, 15 in the United States and seven in Europe. Approximately two‐thirds of subjects were enrolled at U.S. sites and one‐third at European sites. Approval from each study site's institutional ethics committee or appropriate regulatory body was obtained as was informed consent from each study subject or surrogate. Patients were eligible for inclusion if they were ≥18 years of age and presented to an ED or acute health care facility with a GCS of 9–15 (mild to moderate TBI) after a traumatically induced, nonpenetrating head injury resulting from an external force. Patients were enrolled if they underwent noncontrast head CT scan as part of their clinical care, had blood sampling done within 12 h of injury, and provided informed consent. Subjects were excluded if the time of injury could not be determined, if head CT scanning was not performed, if venipuncture was not feasible, or if informed consent was not obtainable. Details regarding patient identification, enrollment, and outcome determination can be found in the ALERT‐TBI study.^11^ The study of plasma specimen testing with the i‐STAT Alinity and TBI plasma cartridge was conducted in compliance with the clinical protocol. Institutional review board (IRB) approval was obtained by each testing site's IRB.

### Measurements

#### Blood sample handling

Serum and plasma specimens prospectively collected during the ALERT‐TBI trial were stored locally at –80°C and then shipped on dry ice to commercial biospecimen storage facilities, where they were again stored at –80°C. Serum samples were analyzed for GFAP and UCH‐L1 using the ELISA‐based BTI. Plasma samples were banked and then later shipped on dry ice to the i‐STAT Alinity and TBI plasma study clinical testing sites. These specimens had not been thawed prior to use in the i‐STAT Alinity and TBI plasma study. The stability of fresh and frozen plasma specimens was established as part of the FDA submission process. These frozen and deidentified plasma samples were tested at three clinical sites: Kentucky Clinical Trials Laboratory, Baylor Scott & White Healthcare, and Penn State Milton S. Hershey Medical Center. Each site tested 647 specimens. Specimens were thawed and tested using i‐STAT TBI plasma cartridges and i‐STAT Alinity instruments.

#### Rapid test

The i‐STAT TBI plasma test is a panel of in vitro diagnostic plasma quantitative measurements of GFAP and UCH‐L1 and a semiquantitative interpretation of test results derived from a combination of these measurements. The i‐STAT TBI plasma test consists of a single‐use test cartridge (i‐STAT TBI plasma test cartridge) that functions with the i‐STAT Alinity system, a portable in vitro diagnostic test system (Data S1, Figure S1, available as supporting information in the online version of this paper, which is available at http://onlinelibrary.wiley.com/doi/10.1111/acem.14366/full). The i‐STAT TBI plasma test cartridge consists of immunoassays for GFAP and UCH‐L1, which are evaluated simultaneously from a single plasma sample. In this study, each plasma specimen was thawed, aliquoted, and centrifuged at 10,000 RCF for 10 min. Approximately 20 µL was then pipetted into the sample well of the i‐STAT TBI plasma test cartridge, which was then inserted into the i‐STAT Alinity system. Sample analysis takes 15 min and concentrations of the two biomarkers are displayed on the analyzer screen. The reportable range for GFAP is 30–10,000 pg/ml and for UCH‐L1 is 200–3200 pg/ml. For the FDA cleared i‐STAT TBI test, the analyzer will not display a value for measurement beyond the reportable range. The estimated lower limit of quantification was 23 pg/ml for GFAP and 70 pg/ml for UCH‐L1. For both assays, the interrun coefficient of variation was less than 10%.

### Outcomes

The primary outcome was acute TII on head CT imaging as determined by two board certified neuroradiologists and adjudicated by a third when needed. CT‐positive was defined as the presence of any of the following intracranial injuries: acute epidural hematoma, acute subdural hematoma, intraventricular hemorrhage, parenchymal hemorrhage/contusion, petechial hemorrhage/bland sheer injury, subarachnoid hemorrhage, brain edema/herniation, and ventricular compression/trapping. CT findings were also categorized as potentially in need of neurosurgical intervention (“neurosurgically manageable”), defined as an acute epidural hematoma >30 cm^3^;^17^ acute subdural hematoma with a thickness >10 mm or a midline shift 5 mm;^18^ a parenchymal contusion > 50 cm^3^; or a frontal/temporal contusions > 20 cm^3^ with midline shift of ≥5 mm or cisternal compression.^19^

### Data analysis

The GFAP and UCH‐L1 concentrations measured by the i‐STAT TBI plasma test were used to report a test interpretation of either “elevated” or “not elevated.” On the i‐STAT Alinity instrument, the test interpretation is displayed on the first page and the second page shows the quantitative results (Figure S1). The i‐STAT TBI plasma test reports elevated if the concentration of one or both proteins is at or above its respective cutoff. The i‐STAT TBI plasma test reports not elevated if the concentrations of both GFAP and the UCH‐L1 are below their respective cutoffs. The cutoffs were determined prior to the study and designed to maximize sensitivity and negative predictive value (NPV). The cutoff for GFAP was 30 pg/ml and for UCH‐L1 was 360 pg/ml. Additional methods describing the derivation of cutoffs are available in Data S1. Dichotomous i‐STAT TBI test interpretations (elevated/not elevated) were correlated to presence or absence of CT‐detected intracranial injury to determine the primary indicators of accuracy, sensitivity, and NPV. The following indicators of accuracy were also determined: specificity, positive predictive value (PPV), likelihood ratio positive, and likelihood ratio negative. Confidence intervals (CIs) for sensitivity, specificity, NPV, and PPV were calculated using the Wilson score method,^20^ while CIs for likelihood ratios were calculated using the Miettinen‐Nurminen score method.^21^ Risk ratios with Haldane's correction^22^ were used to compare the proportion of CT‐positive subjects in those with protein concentrations in the upper 25th, 10th, and 5th percentiles to those below the prespecified cutoff. All analyses were performed using SAS v. 9.4 (SAS Institute).

The total sample size of this study was constrained by the number of archived specimens that met the subject and specimen eligibility requirements. Minimum sample size estimates were determined using the Wilson score test based on the allowable width of the 95% CI for a proportion. Assuming 95% clinical sensitivity with lower bound of the Wilson score 95% CI no less than 90%, the sample size was estimated to be a minimum of 110 CT‐positive subjects.

## RESULTS

### Characteristics of study subjects

**FIGURE 1:**
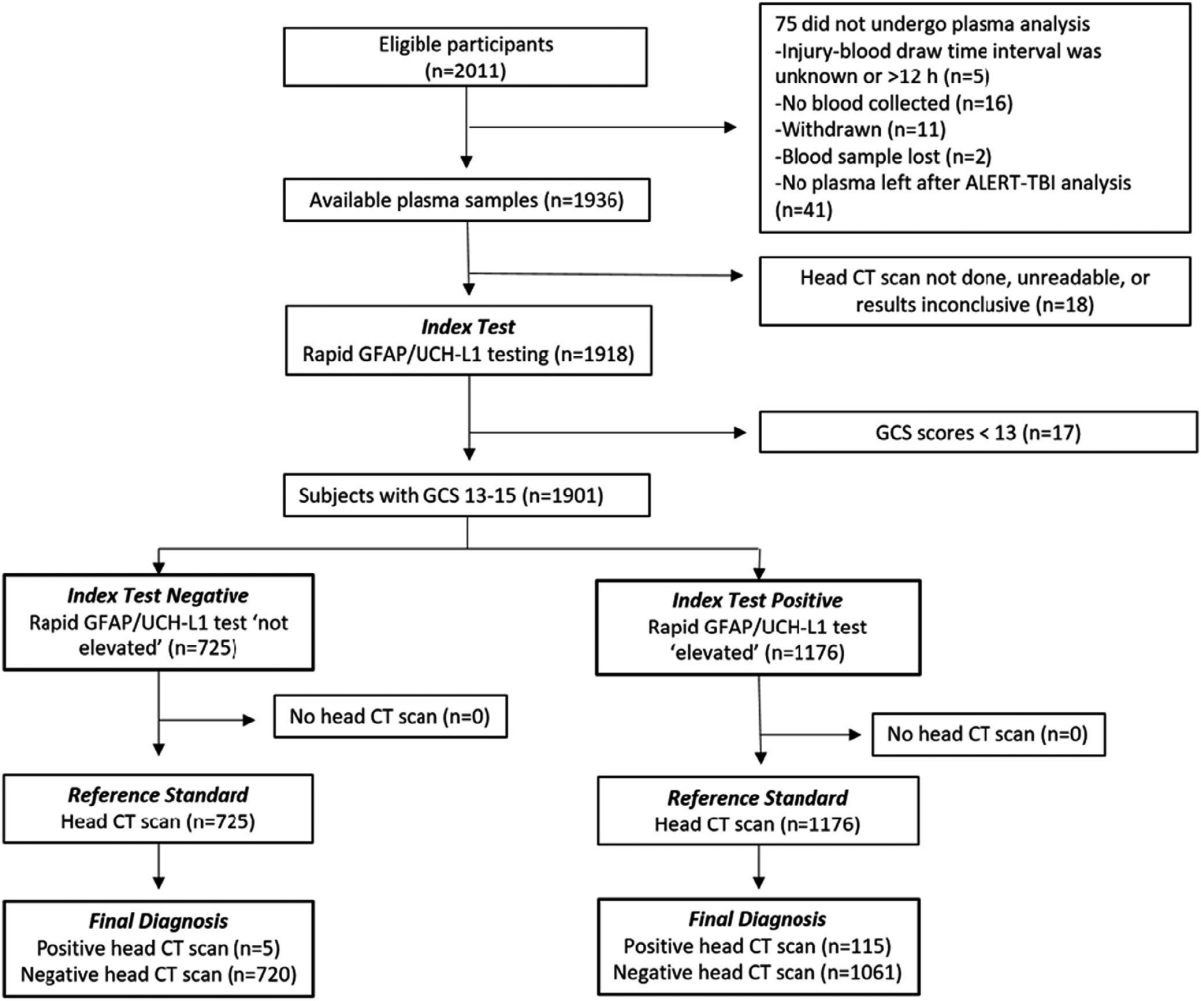
STARD study flow diagram. GCS, Glasgow Coma Scale; GFAP, glial fibrillary acidic protein; UCH‐L1, ubiquitin carboxyl‐terminal hydrolase L1

Of the 2011 subjects enrolled in the ALERT‐TBI study, 1936 had plasma specimens available for testing on the rapid assay platform. Plasma samples from 75 subjects were unavailable because the subject withdrew, did not have blood drawn, or did not consent to future testing, as shown in Figure 1. Of the 1936 subjects with available plasma specimens, 18 had missing or unreadable head CT scans, and thus their plasma samples were not analyzed. The plasma samples from 17 subjects with a Glasgow Coma Scale (GCS) score of 9–12 were analyzed but their results were not presented because this GCS level was considered outside the intended use population (mTBI, GCS 13–15) of the rapid test. The remaining 1901 subjects with a plasma specimen, valid head CT results, and GCS 13–15 were the focus of the current study.

Mild TBI subjects were predominantly Caucasian with a mean (±SD) age of 49.1 (±20.99) years and just over half of the cohort was male (Table 1). Subjects were most commonly injured in a fall or motor vehicle collision, with 42.2% reporting a loss of consciousness (LOC). Of the 1901 mTBI subjects, 1879 (98.8%) had a GCS of 14–15 (Table S1), and 1789 (94.1%) had a GCS of 15 (Table 1). Blood was drawn a median of 3.1 h after injury; 308 of 1789 subjects with a GCS of 15 (17.2%) had blood drawn within 2 h of injury. A total of 120 mTBI subjects (6.3%) had a TII on head CT. Five subjects (0.3%) had neurosurgically manageable lesions. Common head CT findings were subarachnoid hemorrhages (59.2%), acute subdural hematomas (47.5%), and parenchymal hematomas (20.0%). There were no deaths among the study subjects.

**TABLE 1:** Demographic and presenting features of subjects

Characteristics	Subjects with GCS 13–15 (*n* = 1901)	Subjects with GCS 15 (*n* = 1789)	Subjects with GCS 15 and blood collected within 2 h of injury (*n* = 308)
Age (years)	49.1 (±20.99) [18–98]	49.0 (±21.00) [18–98]	55.0 (±22.4) [18–95]
Male sex	1075 (56.5)	1005 (56.2)	184 (59.7)
Race/ethnicity			
White	1343 (70.6)	1254 (70.1)	273 (88.6)
Black or African American	499 (26.2)	477 (26.7)	26 (8.4)
Other/unknown race	70 (3.7)	54 (3.0)	9 (2.9)
Hispanic	90 (4.7)	87 (4.9)	8 (2.6)
GCS score in study site			
9–12	—	—	
13	22 (1.2)	—	
14	90 (4.7)	—	
15	1789 (94.1)	1789 (100)	308 (100)
Mechanism of injury			
Acceleration/deceleration	395 (20.8)	383 (21.4)	27 (8.8)
Motor vehicle collision	578 (30.4)	570 (31.9)	54 (17.5)
Pedestrian struck by vehicle	67 (3.5)	66 (3.7)	8 (2.6)
Fall	987 (51.9)	913 (51.0)	198 (64.3)
Explosion	3 (0.2)	3 (0.2)	1 (0.3)
Assault	179 (9.4)	175 (9.8)	14 (4.5)
Sports injury	47 (2.5)	44 (2.5)	12 (3.9)
Other	53 (2.8)	50 (2.8)	12 (3.9)
Unknown	7 (0.4)	5 (0.3)	2 (0.6)
LOC/PTA			
LOC	803 (42.2)	742 (41.5)	115 (37.3)
PTA	627 (33.0)	566 (31.6)	114 (37.0)
Both LOC and PTA	469 (24.7)	417 (23.3)	62 (20.1)
Neither LOC nor PTA	892 (46.9)	860 (48.1)	121 (39.3)
Unknown	25 (1.3)	18 (1.0)	9 (2.9)
Intoxicated with alcohol or drugs			
Yes	402 (21.2)	367 (20.5)	246 (79.9)
No	1499 (78.9)	1422 (79.5)	62 (20.1)
Head CT scan			
Traumatic injury on head CT	120 (6.3)	94 (5.3)	10 (3.2)
No traumatic injury on head CT	1781 (93.7)	1695 (94.8)	298 (96.8)
Rapid test results			
Hours from injury to blood draw	3.1 [0.3–11.9] (2.3–4.0)	3.2 [0.3–11.9] (2.3–4.0)	1.5 [0.3–2] (1.5–1.8)
GFAP (pg/ml)	36 [0–6856] (17–86) [*n* = 1898]	35 [0–5701] (16–84) [*n* = 1786]	36 [0–1200](19–75) [*n* = 308]
UCH‐L1 (pg/ml)	209 [0–6089] (112–400) [*n* = 1900]	204 [0–5796] (109–379) [*n* = 1789]	298 [11–5796] (1814–582) [*n* = 308]
Positive test	1176 (61.9)	1089 (60.9)	202 (65.6)

Note

Data are reported as mean (±SD), *n* (%), or median [range] (IQR).

Abbreviations: GFAP, glial fibrillary acidic protein; LOC, loss of consciousness, PTA, posttraumatic amnesia; UCH‐L1, ubiquitin carboxyl‐terminal hydrolase L1.

### Main results

**FIGURE 2:**
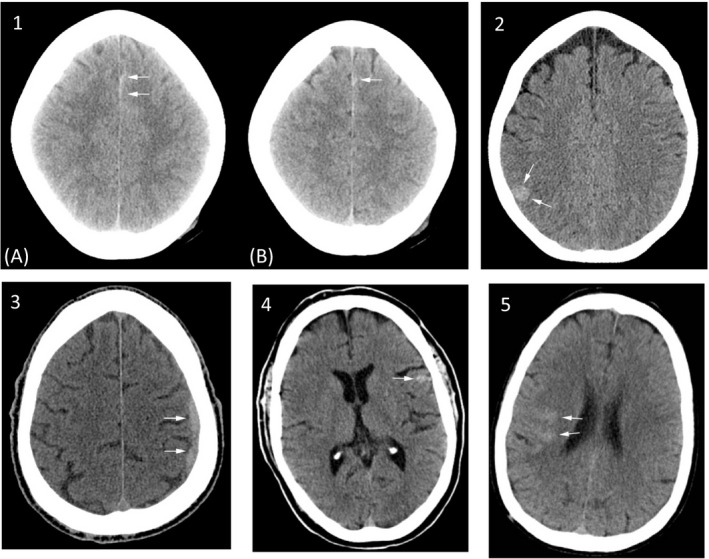
Head CT images of false‐negative subjects. Noncontrast head CT images of five false‐negative subjects. Subject 1: (A) and (B) show focal subarachnoid hemorrhage in the anterior, paramedian frontal sulci. Subject 2: Focal area of hyperdensity in the posterior right parietal lobe, suggestive of a focal hemorrhagic cortical contusion, which was subsequently found on MRI to represent a cavernous malformation. On lower slices (not shown), there is a suggestion of some lower attenuation edema which marginates the contusion. Subject 3: Subdural hemorrhage along the left lateral hemisphere, overlying the frontal and parietal lobes with minimal local mass effect on the brain parenchyma. Subject 4: Left frontal subarachnoid hemorrhage. Subject 5: Right temporal subarachnoid hemorrhage (images for subjects 1–3 reprinted with permission from *Lancet Neurology*)

**FIGURE 3:**
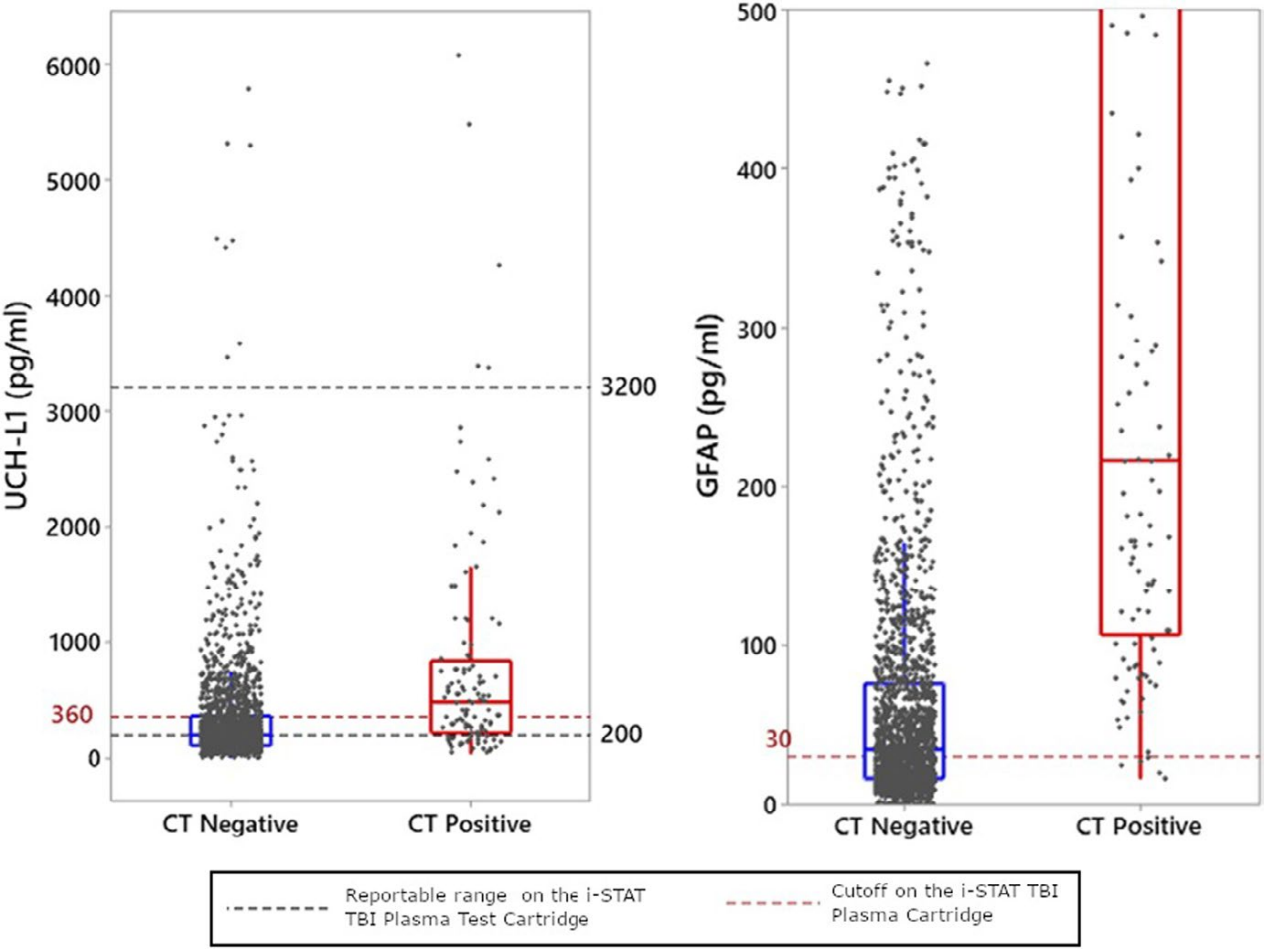
Distribution of GFAP and UCH‐L1 concentrations in subjects with GCS 13–15. GCS, Glasgow Coma Scale; GFAP, glial fibrillary acidic protein; UCH‐L1, ubiquitin carboxyl‐terminal hydrolase L1

Of 1901 mTBI subjects, 1176 (61.9%) had an elevated test and 725 had a not elevated test. Among those with an elevated test, 115 had a positive head CT scan, while among those with a not elevated test, five had a positive scan (Figure 1). The rapid test, thus, had a sensitivity of 0.958 (95% CI = 0.906 to 0.982), specificity of 0.404 (95% CI = 0.382 to 0.427) and NPV of 0.993 (95% CI = 0.985 to 0.997) for acute TII (Figure S2, Table 2). Five subjects had false‐negative test results; four had a presenting GCS of 15 and three had GFAP levels within 20% of the 30 pg/ml cutoff (Table 3). Of the five false‐negative subjects, three had small SAH, one had a small SDH, and one had a parenchymal contusion (Figure 2). None were neurosurgically manageable. Subjects with GFAP or UCH‐L1 concentrations in top 5%, 10%, and 25% of tested subjects were more likely to be CT‐positive compared to subjects with concentrations below their respective prespecified cutoffs for GFAP (30 pg/ml) and UCH‐L1 (360 pg/ml), as shown in Table 4. For both GFAP and UCH‐L1, median values were higher among CT‐positive subjects than from CT‐negative subjects (Figure 3).

**TABLE 2:** Performance of rapid UCH‐L1/GFAP test for predicting acute traumatic intracranial injury on head CT scan

Performance characteristic	Subjects with GCS 13–15 (*N* = 1901)	Subjects with GCS 15 (*n* = 1789)	Subjects with GCS 15 and blood collected within 2 h of injury (*n* = 308)
Sensitivity	95.8 (90.6–98.2)	95.7 (89.6–98.3)	100 (72.3–100)
Specificity	40.4 (38.2–42.7)	41.1 (38.7–43.4)	35.6 (30.4–41.2)
Positive predictive value	9.8 (8.2–11.6)	8.3 (6.8–9.8)	5.0 (2.7–8.9)
Negative predictive value	99.3 (98.4–99.7)	99.4 (98.5–99.8)	100 (96.5–100)
Positive likelihood ratio	1.61 (1.51–1.69)	1.63 (1.51–1.71)	1.52 (1.19–1.66)
Negative likelihood ratio	0.10 (0.04–0.23)	0.10 (0.04–0.26)	0.00 (0.00–0.63)

Note

Data are reported as % (95% CI).

Abbreviations: GCS, Glasgow Coma Scale; GFAP, glial fibrillary acidic protein; UCH‐L1, ubiquitin carboxyl‐terminal hydrolase L1.

**TABLE 3:** Characteristics of false‐negative subjects (*n* = 5)

Sex	Age (years)	Time from injury (h)	GCS	GFAP (pg/ml)	UCH‐L1 (pg/ml)	Head CT findings^b^
Male	62	8.9	15	16	84	Acute SDH
Female	49	5.9	15	24^a^	94	SAH
Female	43	3.5	15	19	58	Parenchymal hematoma
Male	41	3.3	15	26^a^	82	SAH
Male	44	2.9	13	28^a^	184	SAH

Abbreviations: GCS, Glasgow Coma Scale; GFAP, glial fibrillary acidic protein; SAH, subarachnoid hemorrhage; SDH, subdural hematoma; UCH‐L1, ubiquitin carboxyl‐terminal hydrolase L1.

^a^ Value is within 20% of the 30 pg/ml cutoff.

^b^ No head CT findings were neurosurgically manageable injuries (see Figure 2) for CT images.

For the subset of mild TBI subjects with a GCS of 15, the rapid test had a sensitivity of 0.957 (95% CI = 0.896 to 0.983), specificity of 0.411 (95% CI = 0.387 to 0.434) and NPV of 0.994 (95% CI = 0.985 to 0.998) for acute TII. For the subset of GCS 15 subjects having blood drawn within 2 h of injury, the rapid test had a sensitivity of 1.00 (95% CI = 0.723 to 1.00), specificity of 0.356 (95% CI = 0.304 to 0.412), and NPV of 1.00 (95% CI = 0.965 to 1.00) for acute TII (see Table 2, Figure S2, and Table S3). Subjects with GCS 13–14 had similar test performance (Table S2).

## DISCUSSION

The results of this study suggest that, when applied to mTBI patients (GCS 13–15) in whom a head CT is felt to be clinically indicated, a test combining GFAP and UCH‐L1 concentrations measured on a portable, hand‐held device can detect acute TII with a high sensitivity (95.8%). This test was also highly sensitive when applied to the subgroup of patients with a presenting GCS of 15 and to those with GCS of 15 having blood drawn within 2 h of injury. Importantly, the performance characteristics of the i‐STAT TBI plasma test were similar to those for the lab‐based and FDA‐cleared BTI panel using serum and different cutoff values.^11^ In that study, the sensitivity and specificity among those with a GCS 14–15 were 0.973 and 0.367, respectively. However, in contrast to the i‐STAT Alinity, which is easy to use and provides rapid results, the BTI requires labor‐intensive sample preparation, long processing times, and substantial logistical support; these characteristics limit the utility of this platform for acute evaluation of patients.

**TABLE 4:** Risk of acute traumatic injury on head CT scan in subjects with GFAP and UCH‐L1 concentrations in the upper 25th, 10th, and 5th percentiles compared to those below cutoff

Protein	Upper percentile (concentration)	Risk ratio with Haldane's correction applied (95% CI)
UCH‐L1	5th (>1231.5 pg/ml)	28.2 (11.7–79.4)
	10th (>758.5 pg/ml)	23.8 (10.3–65.4)
	25th (>400 pg/ml)	18.4 (8.2–49.6)
GFAP	5th (>372 pg/ml)	57.1 (25.4–154.5)
	10th (>202 pg/ml)	43.2 (19.3–116.0)
	25th (>86 pg/ml)	26.4 (11.9–70.6)

Abbreviations: GCS, Glasgow Coma Scale; GFAP, glial fibrillary acidic protein; UCH‐L1, ubiquitin carboxyl‐terminal hydrolase L1.

For patients seeking care for potential mild to moderate TBI in the ED, CT scan continues to be the imaging modality of choice. There is general agreement, however, that CT scans for mTBI are overutilized and many are avoidable.^3^, ^5^, ^8^ As a result, attempts have been made to reduce head CT use without a consequential increase in missed intracranial injury. The development of a variety of clinical support tools such as CDRs,^23^, ^24^, ^25^, ^26^ published guidelines,^1^, ^27^ and Choosing Wisely campaigns^28^, ^29^ have achieved limited success in reducing CT use. Nearly two decades after the first CDRs were published, CT use continues to be overutilized in adults.^5^, ^30^ One possible reason for this may be that some CDRs, such as New Orleans Criteria^24^ and the Canadian CT Head Rule,^23^ were designed to be applied to patients with either a LOC or amnesia. Our study, however, used broader inclusion criteria, with 47% of subjects having neither LOC nor amnesia. The fact that our results were significant across a broader range of mTBI patients is a distinct advantage of the biomarker panel and suggests the usefulness of this panel extends to patients outside the limited scope of injury defined by CDR criteria. Determining the added value of biomarkers to CDRs is a fruitful avenue for future research.

When applied to mTBI patients (GCS 13–15) in whom a head CT is felt to be clinically indicated, a not elevated i‐STAT TBI plasma test would have resulted in 725 (725/1901; 38.1%) fewer CT scans while missing only five subjects with a positive head CT scan. Three of the five false‐negative subjects were the same ones missed by the BTI. For patients with a GCS score of 15, potential CT reduction would be similar (700/1789; 39.1%), with four false‐negatives. These findings suggest that this test could eliminate the need for some head CTs and potentially alter the trajectory of increased CT use in a favorable direction. Moreover, high rule‐out accuracy combined with test speed might reasonably be expected to facilitate clinical adoption of this test as an aid to head CT decision making in busy EDs where waits for imaging contribute to overcrowding and reduced patient throughput.^12^, ^13^ However, clinical implementation has yet to be specifically studied. Because the test had low to moderate specificity, it has potential to increase CT utilization if used in low‐risk head‐injured patients in whom a CT scan is not otherwise indicated.

While a not elevated i‐STAT TBI plasma test result can have a powerful impact on conserving ED resources and limiting unnecessary radiation exposure, the clinical utility of an elevated test result may seem less apparent. Although an elevated test result is an indication to continue the care pathway toward obtaining a head CT scan, our results show that only 9.8% of study subjects with an elevated test had an acute TII on head CT scan. However, in that 9.8%, the degree of biomarker elevation may be a predictor of unfavorable neurologic outcome in the short term. For example, Lewis et al.^31^ found that GFAP and UCH‐L1 concentrations obtained within 6 h of injury were significantly higher in CT‐positive patients with neurologic deterioration and/or the need for neurosurgical intervention within 24 h of injury compared to CT‐positive patients with favorable outcome.

The relatively modest specificity of the i‐STAT TBI plasma test may be due to its ability to predict subtle TII visible only on MRI scan. Two studies examining GFAP concentrations in mild TBI patients with normal head CT scans found the highest concentrations among those with traumatic injury (usually microhemorrhage) on MRI, followed by those with normal MRIs, then by orthopedic controls, and finally by healthy controls who had the lowest values.^14^, ^32^ The AUCs for GFAP predicting a traumatic injury on MRI in these two studies ranged from 0.777 to 0.803, suggesting that higher concentrations of GFAP were good predictors of TII seen only on MRI. Further research with these biomarkers is under way to determine whether they can be used to predict TII only visible on MRI.

In this study, the cutoffs used to define a not elevated and elevated i‐STAT TBI plasma test result were designed to maximize the accuracy for ruling *out* injuries on head CT scan, not for ruling them *in*; the former requiring high sensitivity and NPV. This strategic decision was based on the needs of ED providers who require an accurate rule‐out test to safely discharge head‐injured patients without a head CT scan. However, our results suggest the rule‐in potential of the i‐STAT TBI plasma test among subjects with GFAP or UCH‐L1 concentrations in top quartile of tested subjects. These subjects were anywhere from 18 to 57 times more likely to have a CT‐detected acute TII compared to those with concentrations below their respective prespecified cutoffs. Although using the i‐STAT TBI plasma test to rule in the need for head CT scanning is outside the FDA indication for use, the PPV accuracy of the i‐STAT TBI plasma test is worthy of further investigation.

### Limitations

This study has several limitations. Only patients in whom head CT scanning was performed for clinical reasons were included, potentially biasing the sample in terms of severity. However, our CT‐positive rate (6.3%) is consistent with prior studies suggesting the study population to be representative.^1^, ^33^ The predictive accuracy was based on CT findings rather than actual clinical outcomes. We categorized CT findings potentially requiring neurosurgical intervention based on guidelines recommending intervention but do not have data on which participants actually required neurosurgical intervention. Our results were produced using banked plasma samples that underwent a single freeze–thaw cycle. Fresh plasma samples were not tested in this study. We did not compare the diagnostic performance of the rapid test to its component proteins. The wide CIs around the point estimate for test sensitivity in subjects with a GCS 15 having blood drawn within 2 h likely reflect the small sample size (*n* = 308). Other study limitations are the same as those described in parent ALERT‐TBI study^11^ and are briefly summarized here. The impact of extracranial injuries on test performance was not evaluated, and test performance was not compared to a CDR. We did not attempt to improve the test's diagnostic accuracy by adding other clinical variables. Because a methodological strength of this study was the use of prespecified marker cutoffs, we chose not to explore alternative cutoffs.

## CONCLUSIONS

In conclusion, the glial fibrillary acidic protein and ubiquitin carboxyl‐terminal hydrolase L1 biomarker panel results obtained using the i‐STAT Alinity and traumatic brain injury plasma test had high sensitivity, but limited specificity, for prediction of acute traumatic intracranial injury, comparable to lab‐based platforms. When used within 12 h of injury among adult mild traumatic brain injury patients in whom a computed tomography scan is felt to be clinically indicated, this test has the potential to reduce computed tomography use. The impact of this test on actual head computed tomography utilization has yet to be determined. The speed, portability, and high accuracy of this test may facilitate clinical adoption of brain biomarker testing as an aid to head computed tomography decision making in EDs.

## CONFLICT OF INTEREST

JJB is on the advisory board and has research support from Abbott, is on the Q30 advisory board, and has research support from BrainScope. RDW has been a paid presenter for Abbott sponsored programs. CAJ, RC, TM, SAD, HZ, and BM are employees of Abbott. SAD is a shareholder of Abbott.

## AUTHOR CONTRIBUTIONS

Jeffrey J. Bazarian, Robert D. Welch, Krista Caudle, Raj Chandran, Tamara McCaw, Hongwei Zhang, and Beth McQuiston were responsible for study concept and study design. Jeffrey J. Bazarian and Robert D. Welch were responsible for acquisition of data. Raj Chandran was responsible for primary statistical analyses. All authors contributed to data analysis and/or interpretation. Jeffrey J. Bazarian, Robert D. Welch, and Tamara McCaw were responsible for drafting the manuscript. Craig A. Jeffrey was the technical lead for iSTAT TBI cartridge development. Krista Caudle was responsible for risk mitigation. Jeffrey J. Bazarian and Robert D. Welch were investigators and authored the manuscript for the original ALERT‐TBI trial. All authors contributed substantially to critical revisions related to important intellectual content. All authors have approved of this submission. Jeffrey J. Bazarian takes primary responsibility for the paper as a whole.

## Supporting information

Figure S1

Figure S2

Data S1
