# Supplementary figures and images for: Accuracy of a rapid glial fibrillary acidic protein/ubiquitin carboxyl‐terminal hydrolase L1 test for the prediction of intracranial injuries on head computed tomography after mild traumatic brain injury
Source: Acad Emerg Med. 2021 Sep 7;28(11):1308–17. doi: 10.1111/acem.14366 (PMC9290667; doi:10.1111/acem.14366)

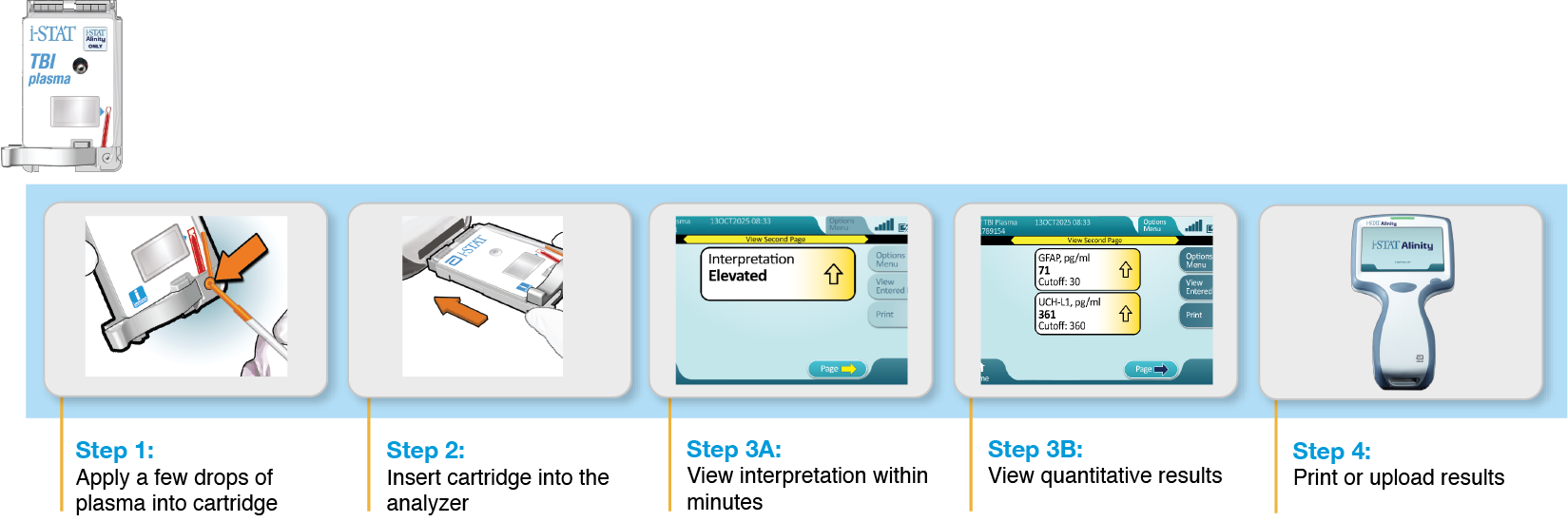

Supplement: Supplementary file 1 — Figure S1 [file ACEM-28-1308-s001.docx]
