# Supplementary material for: Accuracy of a rapid glial fibrillary acidic protein/ubiquitin carboxyl‐terminal hydrolase L1 test for the prediction of intracranial injuries on head computed tomography after mild traumatic brain injury
Source: Acad Emerg Med. 2021 Sep 7;28(11):1308–17. doi: 10.1111/acem.14366 (PMC9290667; doi:10.1111/acem.14366)
Supplement: Supplementary file 3 — Data S1 [file ACEM-28-1308-s003.docx]

**SUPPLEMENTAL MATERIAL**

Method of GFAP and UCH-L1 cut-off derivation

Cutoffs were derived using specimens from the Transforming Research and Clinical Knowledge in Traumatic Brain Injury (TRACK-TBI) Phase I study (Version 17).  There were 420 subjects meeting the inclusion criteria of age >18 years, GCS 13-15, and time from injury to baseline blood draw less than or equal to 12 hours.  The TRACK Phase I study also required subjects to exhibit one or more of the following for inclusion: loss of consciousness, alteration of consciousness, post-traumatic amnesia and/or focal neuro deficits. The observed CT positive prevalence was 18% (78/420).  A ten-fold cross validation with bootstrapping approach was used for cutoff establishment. Cross validation is essential to determining an unbiased result, that is more reflective of the true population. The incorporation of bootstrapping provides control and checks of the stability of the results obtained.

Cutoff Selection Method Steps

1. Draw a random sample of size n WITH REPLACEMENT. (Bootstrapping)

2. Partition each sample from (1) above randomly into 10 equal blocks. (k-fold cross-validation)

3. Use blocks 1 thru 9 (training set) to determine cutoffs for GFAP and UCH-L1

• Consider performance based on cutoffs from all possible biomarker combination cutoffs for the analytical  measuring ranges of both GFAP and UCH-L1.

• Criteria: Adj. NPV >= 98.5% and Sensitivity >= 94%

4. Use the cutoffs from (3) above to calculate the median cutoff

5. Use the median cutoff from (4) above to evaluate the performance on the test set (i.e. the 10th block)

6. Repeat (2) thru (5) above until all 10 combinations of training and test sets are analyzed (i.e., used blocks 1 through 8 and block 10 as the training set, and block 9 as the test set).

7. Result is 10 different GFAP and UCH-L1 median cutoff value combinations

8. Use the results from (7) above to calculate the median cutoff

9.  Repeat (1) thru (8) an additional 500 times, total of 500 cutoff combinations generated

10. Use the results from (9) to calculate the median cutoff for GFAP and UCH-L1

**Supplementary Table 1**: Demographic and Presenting Features of Subjects with GCS 14-15

| **Characteristic** | **Subjects with GCS 14-15**  (n=1879) |
| --- | --- |
| Age, mean yrs (SD)[range] | 49.0 (21.00)  [18, 98] |
| Male sex, No. (%) | 1062 (56.5) |
| Race/ethnicity, No. (%) |  |
| White | 1323 (70.4) |
| Black or African American | 498 (26.5) |
| Other/unknown race | 69 (3.7) |
| Hispanic | 89 (4.7) |
| GCS score in study site, No. (%) |  |
| 9-12 | -- |
| 13 | -- |
| 14 | 90 (4.8) |
| 15 | 1789 (95.2) |
| Mechanism of Injury, No. (%) |  |
| Acceleration/Deceleration | 391 (20.8) |
| Motor Vehicle Accident | 573 (30.5) |
| Pedestrian Struck by Vehicle | 67 (3.6) |
| Fall | 971 (51.7) |
| Explosion | 3 (0.2) |
| Assault | 179 (9.5) |
| Sports Injury | 47 (2.5) |
| Other | 53 (2.8) |
| Unknown | 7 (0.4) |
| LOC/PTA, No. (%)* |  |
| LOC | 790 (42.0) |
| PTA | 616 (32.8) |
| Both LOC and PTA | 458 (24.4) |
| Neither LOC nor PTA | 887 (47.2) |
| Unknown | 21 (1.1) |
| Intoxicated with alcohol or drugs, No. (%) |  |
| Y | 395 (21.0) |
| N | 1484 (79.0) |
| Head CT Scan |  |
| Traumatic injury on head CT, No. (%) | 113 (6.0) |
| No Traumatic injury on head CT, No. (%) | 1766 (94.0) |
| Rapid Test Results |  |
| Hours from injury to blood draw, median,[range], (IQR) | 3.2 [0.3, 11.9]  (2.3-4.0) |
| GFAP, median, [range], (IQR) pg/ml | 36 [0,6856]  (16-86) [n=1876] |
| UCH-L1, median [range], (IQR) pg/ml | 208 [0, 5796]  (111-398) [n=1879] |
| Positive Test, No. (%) | 1,159 (61.7) |

*LOC: loss of consciousness, PTA: post-traumatic amnesia

**Supplementary Table 2**: Performance of rapid UCH-L1/GFAP Test for Predicting Acute Traumatic Intracranial Injury on Head CT Scan in Subjects with GCS 14-15

| \| **Performance Characteristic** \| **Subjects with GCS 14-15** \| \| --- \| --- \| \| Sensitivity, % (95%CI) \| 96.5 (91.3, 98.6) \| \| Specificity, % (95%CI) \| 40.5 (38.3, 42.9) \| \| Positive Predictive Value, % (95%CI) \| 9.4 (7.9, 11.2) \| \| Negative Predictive Value, % (95%CI) \| 99.4 (98.6, 99.8) \| \| Positive Likelihood Ratio (95%CI) \| 1.61 (1.51, 1.69) \| \| Negative Likelihood Ratio (95%CI) \| 0.10 (0.04, 0.23) \| |
| --- | --- | --- | --- | --- | --- | --- | --- | --- | --- | --- | --- | --- | --- | --- |

| **Supplementary Table 3**: Performance of rapid UCH-L1/GFAP Test for Predicting Acute Traumatic Intracranial Injury on Head CT Scan in Subjects with GCS 15 | | | |
| --- | --- | --- | --- |
|  | CT Positive (N=94) | CT Negative (N=1695) |  |
| Test Positive  (N=1089) | 90 | 999 |  |
| Test Negative  (N=700) | 4 | 696 |  |
|  | | | |
