# Supplementary material for: Accuracy of a rapid glial fibrillary acidic protein/ubiquitin carboxyl‐terminal hydrolase L1 test for the prediction of intracranial injuries on head computed tomography after mild traumatic brain injury
Source: Acad Emerg Med. 2021 Sep 7;28(11):1308–17. doi: 10.1111/acem.14366 (PMC9290667; doi:10.1111/acem.14366)
Supplement: Supplementary file 3 [file ACEM-28-1308-s002.docx]

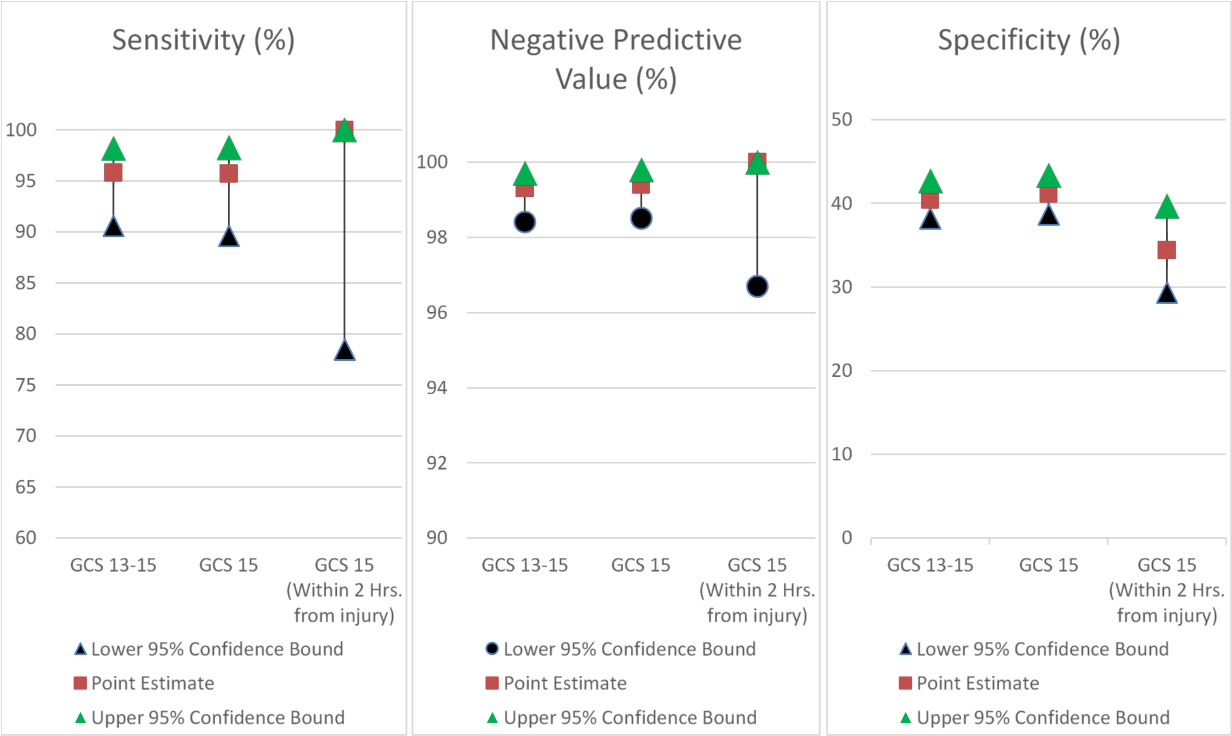

Sensitivity (%)
100
95
90
85
80
75
70
65
60
GCS 13-15
GCS 15
GCS 15 (Within 2 Hrs. from injury)
Lower 95% Confidence Bound
Point Estimate
Upper 95% Confidence Bound
Negative Predictive Value (%)
100
98
96
94
92
90
GCS 13-15
GCS 15
GCS 15 (Within 2 Hrs. from injury)
Lower 95% Confidence Bound
Point Estimate
Upper 95% Confidence Bound
Specificity (%)
50
40
30
20
10
0
GCS 13-15
GCS 15
GCS 15 (Within 2 Hrs. from injury)
Lower 95% Confidence Bound
Point Estimate
Upper 95% Confidence Bound
